# Janus kinases restrain chronic lymphocytic leukemia cells in patients on ibrutinib: Results of a phase II trial

**DOI:** 10.1002/cam4.4378

**Published:** 2021-11-17

**Authors:** David E. Spaner, Yuxuan Luo, Guizhei Wang, Jennifer Gallagher, Hubert Tsui, Yonghong Shi

**Affiliations:** ^1^ Biology Platform Sunnybrook Research Institute Toronto Ontario Canada; ^2^ Department of Medical Biophysics University of Toronto Toronto Ontario Canada; ^3^ Sunnybrook Odette Cancer Center Toronto Ontario Canada; ^4^ Department of Medicine University of Toronto Toronto Ontario Canada; ^5^ Department of Immunology University of Toronto Toronto Ontario Canada; ^6^ Division of Hematological Pathology Sunnybrook Health Sciences Centre Toronto Ontario Canada; ^7^ Department of Laboratory Medicine and Pathobiology University of Toronto Toronto Ontario Canada

**Keywords:** chronic lymphocytic leukemia, glucocorticoids, ibrutinib, interferon, janus kinases, ruxolitinib, TNF

## Abstract

Preclinical observations that killing of chronic lymphocytic leukemia (CLL) cells was dexamethasone (DEX) were enhanced by concomitant inhibition of Bruton’s tyrosine kinase and janus kinases (JAKs) motivated a phase II trial to determine if clinical responses to ibrutinib could be deepened by DEX and the JAK inhibitor ruxolitinib. Patients on ibrutinib at 420 mg daily for 2 months or with abnormal serum β2M levels after 6 months or with persistent lymphadenopathy or splenomegaly after 12 months were randomized to receive DEX 40 mg on days 1–4 of a 4‐week cycle for six cycles alone (three patients) or with ruxolitinib 15 mg BID on days 1–21 of each cycle (five patients). Ruxolitinib dosing was based on a previous phase I trial. Steroid withdrawal symptoms and significantly decreased serum IgG levels occurred in all patients regardless of their exposure to ruxolitinib. A fatal invasive fungal infection was seen in a patient taking DEX without ruxolitinib. Complete responses anticipated with addition of ruxolitinib were not seen. Gene expression studies suggested ruxolitinib had turned off interferon signaling in CLL cells and turned on genes associated with the activation of NFκB by TNF‐α. Ruxolitinib increased blood levels of TNF‐α by cycle 3 and decreased the inhibitory cytokine IL‐10. These results suggest ruxolitinib releases activating signals for CLL cells that persist in patients on ibrutinib. This inhibitory JAK signaling may contribute to the therapeutic activity of ibrutinib. Thus JAK inhibitors provide no added value with ibrutinib for disease control and should be used with caution in CLL patients. Combining glucocorticoids with ibrutinib may increase the risk of serious infects.

## Graphical abstract

Ibrutinib with ruxolitinib and dexamethasone is generally well‐tolerated but provides no benefits over ibrutinib alone. Ruxolitinib blocks inhibitory signals that suppress CLL cells in patients on ibrutinib.

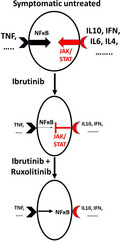

## 1 INTRODUCTION

The Bruton’s tyrosine kinase (BTK) inhibitor ibrutinib has been a major advance for chronic lymphocytic leukemia (CLL) patients but it does not cure as a single agent, outcomes for patients who ultimately progress on it are poor, and strategies to improve its activity are needed.^1^, ^2^ Concomitant inhibition of janus kinases (JAKs) might be a rational approach to deepen clinical responses to ibrutinib^3^, ^4^ as cytokine signaling through JAKs can promote the growth and survival of CLL cells and is not prevented by BTK blockade.^5^, ^6^ Ibrutinib reduces blood levels of many cytokines that are elevated in symptomatic patients but others such as IL‐6 are not changed significantly^6^ and may continue to provide support for CLL cells.^7^, ^8^ By simultaneously removing two major pathways to survival, blocking both BTK and JAK signaling might be expected to increase killing of CLL cells and deepen clinical responses.

To this end, ruxolitinib, a JAK1/2 inhibitor approved for steroid‐refractory acute graft‐versus‐host disease as well as intermediate or high‐risk myelofibrosis and polycythemia vera after an inadequate response or intolerance to hydroxyurea was studied in CLL patients.^9^, ^30^ Ruxolitinib’s toxicity as a single agent in CLL has been characterized in the front‐line setting^3^ and 15 mg twice daily identified as a dose that can be combined safely with 420 mg of ibrutinib over a 7‐month period.^4^ The combination had modest therapeutic effects^4^ but it was hypothesized that the potential advantages of using both ibrutinib and ruxolitinib might be realized by adding a cytotoxic agent as both inhibitors are not particularly toxic to CLL cells.^5^, ^10^

The glucocorticoid dexamethasone (DEX) was chosen because it has significant cytotoxic activity and a well‐known toxicity profile in CLL.^11^ In addition, pre‐clinical studies had demonstrated that CLL cells from many patients that were resistant to DEX‐mediated killing in an in vitro microenvironmental model were killed in the presence of ruxolitinib and ibrutinib but not ibrutinib alone.^10^

A randomized phase II trial was designed to determine if six cycles of DEX combined with ruxolitinib plus ibrutinib could induce complete responses (CRs) and a state of low minimal residual disease (MRD). A control group of patients on ibrutinib treated with six cycles of DEX was used for comparison as ibrutinib alone does not induce CRs in this period of time.^12^ The results are presented below.

## 2 MATERIALS AND METHODS

### 2.1 Study design and participants

This was a single‐center randomized phase II study to determine if ruxolitinib could increase the depth of response of CLL patients to ibrutinib and DEX. Eligible patients had either just commenced ibrutinib at 420 mg daily as first‐line therapy or for relapsed/refractory disease after a run‐in period of 8 weeks to allow initial side effects of ibrutinib to abate or met the criteria of the prior phase I trial^4^ and had been treated with ibrutinib for more than 6 months with: (i) failure of plasma β2M levels to decrease below 2.5 μg/L within 6 months of starting ibrutinib or (ii) persistent lymphocytosis (>5 × 10^6^ cells/L) and splenomegaly (>11.5 cm) or lymphadenopathy (marker node >1.5 cm on CT scans) after 1 year on ibrutinib.^4^ Exclusion criteria included inadequate bone marrow reserve indicated by neutrophils less than 0.75 × 10^9^/L, platelets less than 75 × 10^9^/L without the assistance of growth factors, thrombopoietic factors, or platelet transfusions, or hemoglobin less than 65 g/L despite transfusions.

The study was approved by the Sunnybrook Research Ethics Board and Health Canada and conducted according to the principles of the Declaration of Helsinki and the Guidelines for Good Clinical Practice. All patients provided written informed consent. The trial was registered with ClinicalTrials.gov, number NCT02912754. Studies involving human samples were reviewed and approved by the Sunnybrook Research Ethics Board (PIN 222‐2014).

### 2.2 Procedures

Ibrutinib was continued at 420 mg daily. Patients were randomized to a control arm treated additionally with DEX or an experimental treatment arm involving DEX and ruxolitinib for six cycles of 28 days. DEX was given at 40 mg daily on days 1–4 of each cycle along with antiviral and *Pneumocystis jiroveci* pneumonia prophylaxis and bisphosphonates. Ruxolitinib was used at 15 mg twice daily on days 1–21 of each cycle.^4^

Therapeutic activity was evaluated at the end of treatment (EOT) visit that occurred 30 days after cycle 6 day 1 (C6D1). Based on estimates from in vitro modeling^10^ that meaningful efficacy of ibrutinib, ruxolitinib, and DEX required the ability to induce CRs in 30% of patients while the CR rate with DEX and ibrutinib was likely be 0%, 11 patients were planned to be enrolled in each group to demonstrate significant differences with a power of 0.8 and *α*‐value of 0.05.

The primary endpoint was overall response rate, defined as proportion of subjects with CRs or partial responses (PRs) at EOT, according to the NCI‐WG guidelines on CLL.^13^ Secondary endpoints related to the safety and tolerability of DEX with or without ruxolitinib when combined with ibrutinib.

Patients were monitored by history, physical examination, and blood tests on days 1 and 14 of cycle 1, day 1 of cycles 2–6, and at EOT. Adverse events were graded according to the Common Toxicity Criteria of the National Cancer Institute, Version 4.0. Hematological toxicities were graded according to IWCLL 2008 criteria.^13^ Response assessments by CT scans were performed after six cycles or earlier if indicated clinically. Bone marrow aspirates and biopsies were taken prior to study entry and at EOT only if the circulating lymphocyte count did not indicate persistent disease. Responses were assessed by IWCLL guidelines.^13^

Exploratory endpoints included measurements of plasma cytokines and changes in gene expression in circulating CLL cells. Statistical analysis was mainly descriptive due to the nature of the study.

### 2.3 Cell and plasma preparation

CLL cells were isolated from blood and bone marrow by negative selection and density gradient centrifugation as before.^4^, ^5^ Aliquots were cryopreserved. Plasma was also aliquoted and stored at −80°C.

### 2.4 Serum immunoglobulins, lactate dehydrogenase, and complete blood counts

Serum IgG, IgM, and IgA levels along with lactate dehydrogenase (LDH) were measured by the clinical service laboratory. Mean red cell volumes (MCVs) along with platelet and lymphocyte counts were determined in the clinical hematology laboratory. The results were taken from the patients’ electronic medical records.

### 2.5 Flow cytometry

Blood samples were enumerated by a hematology analyzer Beckman Coulter (BC) (Fullerton, CA) in the clinical hematology laboratory. White blood cells (WBCs) were adjusted to 1 × 10^7^ cells/ml and 100 μl (1 × 10^6^ cells) stained with antibody combinations. Red blood cells were lysed with VersaLyse™ (BC). Samples were run on a 10 color Navios™ (BC) flow cytometer and analyzed with Kaluza™ software (BC). At least 5 × 10^4^ leukocytes were acquired per sample.

### 2.6 Plasma cytokines

Serum TNF‐α and IL‐10 levels were measured by Eve Technologies using Multiplexing LASER Bead Technology.^3^, ^4^ Concentrations were determined from standard curves. Assays were linear between 30 and 1000 pg/ml of cytokine.

### 2.7 RNA‐Seq

Transcript data are summarized in Table S1. Briefly, RNA was extracted from CLL cells purified from five patients on ibrutinib alone and then while on ibrutinib plus ruxolitinib for 3 weeks. RNA was subjected to the PCR‐based AmpliSeq Transcriptome Human Assay, using a Thermo Fisher ion S5xl instrument. The AmpliSeq RNA plug‐in Ion‐torrent server was used to provide initial read numbers per gene and normalization for all 10 samples. From an initial list of 20,812 genes, 12,477 remained after filtering out low expressed genes (less than 10 reads in 10 samples) and the large class of olfactory receptors^14^ prior to gene set enrichment analysis (GSEA) analysis.

### 2.8 Gene set enrichment analysis

Samples on ruxolitinib with ibrutinib were compared to samples on ibrutinib alone by the methods of GSEA (version 4.1.0, Broad Institute).^15^, ^16^ Enrichments were considered significant with a false discovery rate (FDR) <25% and nominal *p* value <1%.

### 2.9 Statistical analysis

Comparisons between two groups of measurements were tested for significance by the Student's or paired *t*‐tests with *p* < 0.05 considered significant. Analysis of variance (ANOVA) with multiple comparisons was conducted to determine the significance of differences between multiple groups.

## 3 RESULTS

### 3.1 Patients

Between 22 August 2019 and 26 May 2020, eight patients were enrolled in the study. Five patients were randomized to the DEX/ruxolitinib arm and three to the DEX only arm. Patient characteristics are described in Table 1. The study was stopped early due to a death of one patient taking DEX only, no evidence of CRs in the patients on ruxolitinib, and identification of a mechanism that predicted further lack of efficacy.

**TABLE 1:** Patient data

Patient no.	Sex	Age	C1D1 lymphs (x10^9^/L)	C1D1 β2M^a^	IGHV^b^	FISH^c^/mutations	Prior treatment	Time on ibrutinib (months)
SDEX/RUX								
JAK3001	M	64	55.9	1.8	M	del(13q)	FCR	2
JAK3003	M	69	2	2.8	NA	del(13q)	FCR	46
JAK3005	F	54	8.2	2.3	U	t12^e^	None	2
JAK3006	M	75	387	3.2	U	del (11q), del (13q), BIRC3 Q484fs*13^f^	None	2
JAK3007	M	69	1.6	1.8	NA	del (17p), del(6q)	BR	15
DEX								
JAK3002	M	72	50	3.5	NA	del (11q), del (13q)	FCR	38
JAK3004	M	62	2.3	2.6	U	Normal	None	7
JAK3008	M	64	1.9	2.4	NA	del(17p), t12	FCR	40

^a^ Normal range: 0.6–2.3 μg/ml.

^b^ M = mutated; U = unmutated; NA = not available.

^c^ Fluorescence in situ hybridization.

^d^ FCR = fludarabine, cyclophosphamide, rituxan; BR = bendamustine, rituxan.

^e^ T12 = trisomy 12.

^f^ Genome analysis was available for this patient from FOUNDATIONONE®HEME panel results obtained on a commercial basis.

### 3.2 Toxicity

All patients complained of steroid withdrawal symptoms following the 4 days of DEX. Three patients required a brief steroid taper on days 5–7 to ameliorate these symptoms. JAK3006 developed new onset atrial fibrillation and JAK3002 experienced a recurrence of previous atrial fibrillation.

**FIGURE 1:**
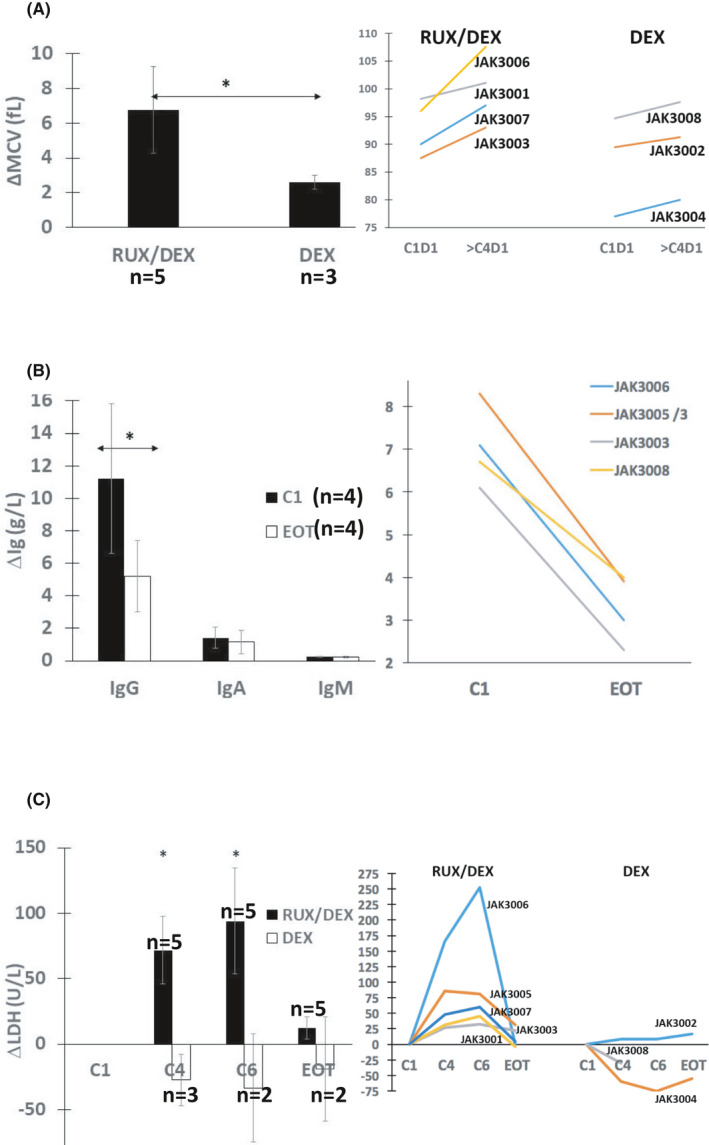
Effect of dexamethasone with or without ruxolitinib on red cell volumes, serum immunoglobulins, and lactate dehydrogenase levels. (A) Mean red cell volumes (MCVs) at C1D1 were subtracted from MCVs taken from the patients’ clinical records at C6D1 or CD4D1 for JAK3008. Averages and standard errors for the two study arms are shown in the left graph with results for individual patients shown on the right. (B) IgG, IgA, and IgM were measured at C1D1 and either EOT for JAK3003, 3005, and 3006 or C4D1 for JAK3008. The other patients were receiving immunoglobulin replacement therapy. Averages and standard errors at these times are shown in the left bar graph with results for IgG levels in individual patients graphed on the right. (C) Serum LDH levels at C1D1 were subtracted from the levels measured at C4D1 for all patients and with JAK3008 excluded at C6D1 and EOT. The averages and standard errors for patients on dexamethasone with or without ruxolitinib are shown in the left bar graph with the results for individual patients shown in the right line graph. *p < 0.05

JAK3001 developed neutropenia that recovered without growth factors after delaying cycle 3 day 1 (C3D1) by 1 week. Anemia did not occur but MCVs in the DEX/ruxolitinib arm increased (Figure 1A), suggesting impaired erythropoiesis as seen before with ruxolitinib as a single agent in CLL.^3^

Four patients (JAK3001, JAK3002, JAK3004, and JAK3007) were receiving immunoglobulin replacement at study entry. Serum immunoglobulin levels were decreased by DEX in the rest, particularly IgG (Figure 1B). Two patients (JAK3003 and JAK3005) were treated with oral antibiotics for upper respiratory tract infections. JAK3008 died from invasive aspergillosis prior to cycle 5.

Serum LDH in patients taking DEX with ruxolitinib increased relative to baseline by cycle 4 and remained high at cycle 6 but returned to baseline at EOT. These changes were not accompanied by other markers of tumor lysis syndrome or observed in patients taking only DEX (Figure 1C).

### 3.3 Clinical responses

Tumor burdens in most patients entering the study were already low (Table 1) and adding ruxolitinib and DEX to ibrutinib had been predicted to induce molecular remissions in at least three of them.^4^, ^10^ However, the best responses were PRs in three patients (JAK3001, JAK3005, and JAK3006) who were on ibrutinib for only 2 months before adding ruxolitinib and DEX and could not be distinguished from simply continuing ibrutinib for this time. The other two patients who completed the course of DEX and ruxolitinib were classed as having stable disease (SD). JAK3002 on DEX alone exhibited a PR at EOT but progressed within 6 months and is now being treated with venetoclax. JAK3004 who also received DEX alone was classed as having SD at EOT.

**FIGURE 2:**
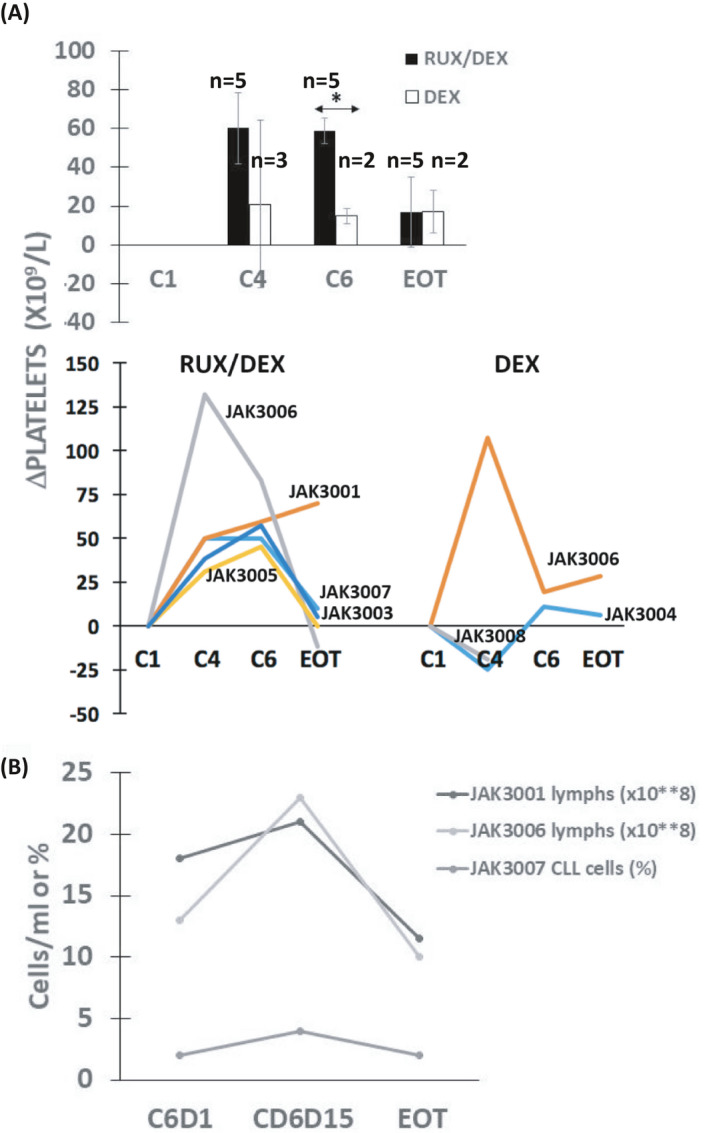
Effect of ruxolitinib and dexamethasone on platelets and lymphocytes. (A) Platelet counts were extracted from the medical records. Differences between platelet numbers at day 1 of the indicated cycles and the count at cycle 1 day 1 (C1D1) were calculated for each patient. Averages and standard errors for patients on dexamethasone with or without ruxolitinib are shown in the bar graph (top) with results for the individual patients in the line graph (bottom). (B) Lymphocyte numbers at each timepoint were taken from the medical records for JAK3001 and JAK3006. Blood from JAK3007 was collected at C6D1, C6D15, and end of treatment (EOT) and percentages of CD5^+^CD19^+^ CLL cells were measured by 10‐color flow cytometry. The results suggest ruxolitinib flushes CLL cells into the circulation despite the presence of dexamethasone. *p < 0.05

Improved hematologic parameters are associated with positive responses to CLL treatments.^13^ Platelet counts increased significantly by cycle 6 in patients taking ruxolitinib and DEX compared to patients treated only with DEX but returned to baseline at EOT (Figure 2A). Ruxolitinib also transiently increased platelets in the phase I trial with ibrutinib,^4^ suggesting this phenomenon was not prevented by DEX.

Ruxolitinib is known to shift residual leukemia cells into the blood as a single agent^3^ and in combination with ibrutinib.^4^ Percentages of CD5^+^CD19^+^ CLL cells, measured by flow cytometry, increased transiently in JAK2007, who otherwise had normal blood lymphocyte counts (Figure 2B). Ruxolitinib also caused a transient increase in lymphocytes that reversed upon stopping it for at least a week in JAK3001 and JAK3006, who had elevated lymphocyte counts consisting mainly of CLL cells (Table 1, Figure 2B). These findings suggested that the ability of ruxolitinib to flush CLL cells out of extravascular microenvironments was not altered by DEX.

### 3.4 Effect of ruxolitinib on gene expression and plasma cytokines

**FIGURE 3:**
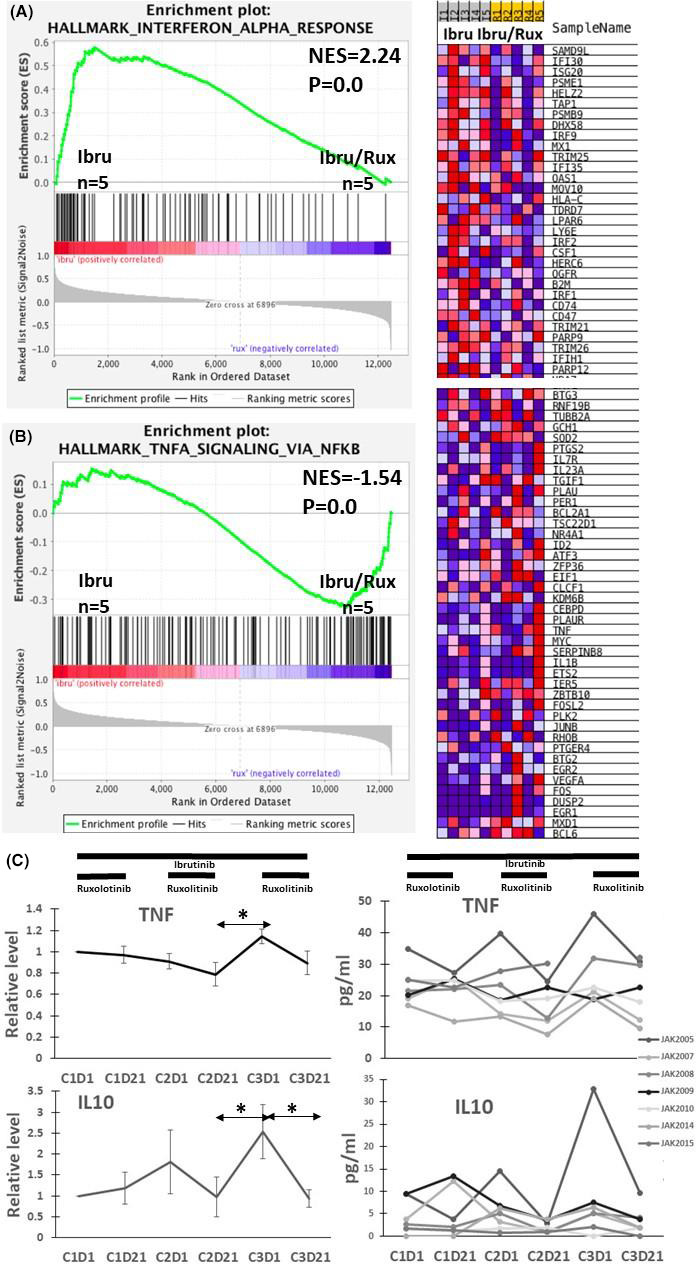
Effect of ruxolitinib on interferon and TNF gene signatures in CLL cells and TNF‐α and IL‐10 protein levels in blood. (A, B) Gene expression was determined in CLL cells purified from five patients on ibrutinib and following addition of ruxolitinib. GSEA enrichment plots depicting significant enrichment of interferon response genes in CLL cells on ibrutinib (A) and TNF signaling response genes in CLL cells also exposed to ruxolitinib (B) are shown on the left with heatmaps of the corresponding leading edge genes on the right. (C) Blood levels of TNF‐α and IL‐10 were measured in 10 patients on ibrutinib for at least 9 months before and after concomitant treatment with ruxolitinib. Ibrutinib was continuous while ruxolitinib was cycled for 3 weeks “on” (indicated by the solid line) followed by a break period of 2 weeks “off.” Results for three consecutive cycles are shown. C1D1 is the beginning of cycle 1 and C1D21 is 3 weeks later when ruxolitinib was held, etc. Individual measurements at each timepoint are shown in the graphs on the right. On the left, values were normalized to the C1D1 value and the averages and standard errors for all patients are shown at each timepoint. *p < 0.05

Low numbers of circulating leukemia cells in many of the trial patients made it difficult to obtain large amounts of purified CLL cells for correlative studies. Messenger RNA was isolated from CLL cells of JAK3001 and JAK3006 while taking ibrutinib before starting DEX and ruxolitinib and 3 weeks later when they were on both ibrutinib and ruxolitinib and DEX had been discontinued for 17 days. RNA from purified CLL cells was also available from three patients in the prior phase I trial^4^ at cycle 1 day 1, before starting ruxolitinib and at cycle 3 day 21, while on both ibrutinib and ruxolitinib. The mRNA from these five patients was analyzed by RNA‐Seq as described in the materials and methods. Gene expression levels for each sample are listed in Table S1. The results from all five patients were pooled for GSEA compared to the 50 Hallmark gene sets of the MSigDB collection (Figure 3A,B).^14^, ^15^

In CLL cells exposed only to ibrutinib in vivo, five gene sets were significantly enriched at a FDR <25% and three were enriched significantly at nominal *p* < 0.01. The top two sets were hallmark interferon (IFN)‐alpha (Figure 3A) and hallmark interferon‐gamma response.

In CLL cells exposed to both ruxolitinib and ibrutinib in vivo, nine gene sets were enriched at a FDR <25% with five enriched at nominal *p* < 0.01. These sets included hallmarks G2‐M checkpoint, E2F targets, and mitotic spindle, associated with a state of cellular activation and cell cycle progression, along with hallmark TNFA signaling via NFκB (Figure 3B). Taken together, these findings suggested ruxolitinib turned off type 1 and 2 IFN signaling and stimulated NFκB activity in CLL cells.

Serum cytokines were not measured in this phase II trial but the GSEA results prompted a re‐examination of cytokine levels in the blood of patients on ibrutinib and ruxolitinib in a phase I trial without DEX.^4^ In the latter trial, ibrutinib was taken continuously at 420 mg daily while ruxolitinib was administered on a discontinuous schedule, consisting of seven 5‐week cycles when it was taken for 3 weeks followed by a rest period of 2 weeks. This “off period” enabled repetitive measurements of ruxolitinib‐mediated effects on plasma cytokines in vivo.^4^, ^5^

The interferon‐stimulated gene products β2M, CXCL10, and IL‐18 were shown before to decrease in patients following each cycle of treatment with ruxolitinib, consistent with inhibition of IFN signaling.^4^, ^5^ IL‐10 is a well‐known negative regulator of NFκB signaling^17^ that has been shown to decline in the blood of patients on ibrutinib after adding ruxolitinib.^4^ Examination of more timepoints indicated IL‐10 levels in patients on ibrutinib also exhibited a cycling behavior in response to ruxolitinib (Figure 3B). TNF‐α was detected in the blood of all patients and increased above baseline by the third cycle of ruxolitinib (Figure 3B).

## 4 DISCUSSION

**FIGURE 4:**
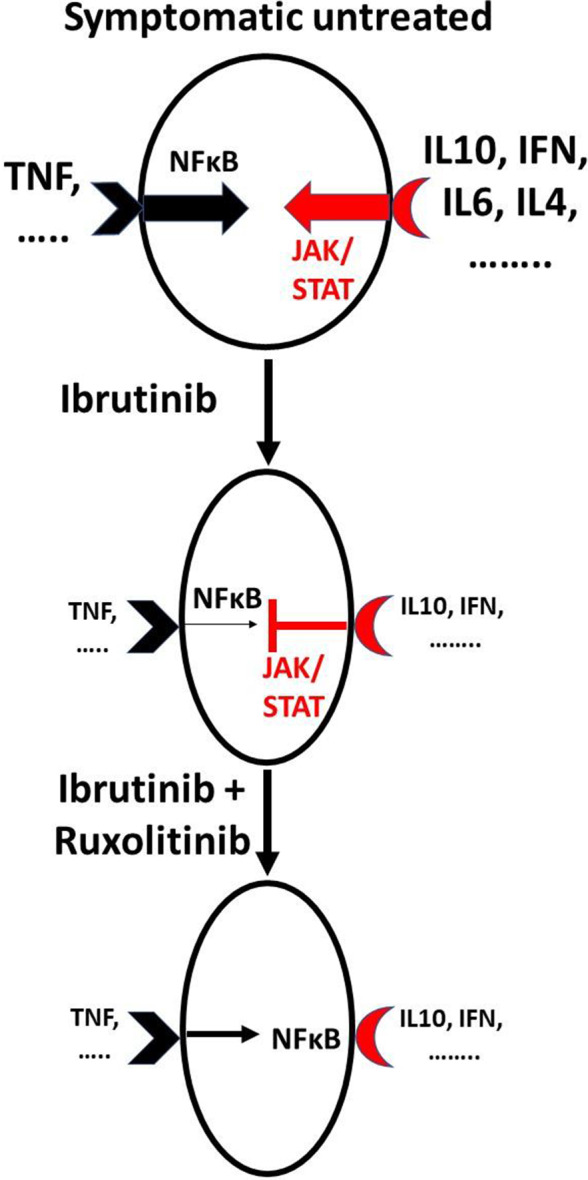
Schema of ruxolitinib and ibrutinib interactions in vivo. A complex network of cytokines that can activate JAK/STAT and NFκB signaling pathways is a feature of symptomatic CLL. Ibrutinib decreases most cytokines but some, particularly type 1 IFN, IFN‐γ, and IL‐10, continue to affect CLL cells and suppress NFκB signaling processes that are not blocked completely by ibrutinib. Ruxolitinib removes the inhibitory activity of these cytokines, allowing increased activity of NFκB that can mediate resistance to cytotoxic agents. Sizes of letters and arrows indicate levels and activity of cytokines and signaling pathways

The number of recruited patients in this trial was low but the absence of responses in five patients treated with ruxolitinib and DEX in addition to ibrutinib (Table 2) is sufficient to conclude that JAK inhibitors provide no added value with ibrutinib for disease control. The small number of patients treated with ruxolitinib was still able to provide the data in Figures 3 and 4 supporting the idea that JAK signaling helps to maintain CLL cells in an inactive state in the presence of ibrutinib and thus may contribute to the mechanism of action of ibrutinib.

**TABLE 2:** Responses

Patient no.	Blood lymphs (x10^9^/L)		Marrow CLL cells (%)		Marker LN (cm)		Spleen (cm)		Response^a^
	C1D1	EOT	C1D1	EOT	C1D1	EOT	C1D1	EOT	
DEX/RUX									
JAK3001	56	11.5	NA	NA	None	None	19.2	14.5	PR
JAK3003	2	1.9	10	4	3.7	3.1	11.3	10.7	SD
JAK3005	8	3	NA	10	2.6	1.2	14	8.3	PR
JAK3006	387	103	NA	NA	3.8	1.4	19.2	15.3	PR
JAK3007	1.5	0.6	0.8	0.2	None	None	12.5	11.9	SD
DEX									
JAK3002	56	13	NA	NA	2.1	1.5	12.5	10.9	PR
JAK3004	2	3	5	5	3	2	17	15	SD
JAK3008	1.9	NA	2	NA	1.7	NA	11.3	NA	Died

Abbreviations: NA, not available; PD, progressive disease; PR, partial response; SD, stable disease.

Ruxolitinib appears to inhibit JAK‐mediated negative regulation of signaling processes that are ongoing in patients on ibrutinib such as from TNF‐α in plasma (Figure 3). CLL cells from JAK3006 also harbored an activating *BIRC3* mutation that would be expected to facilitate non‐canonical NFκB signaling despite the presence of ibrutinib and ruxolitinib (Table 1).^18^ Removal of inhibitory signaling by ruxolitinib apparently drives CLL cells to express gene patterns associated with a more activated state (Figure 3A), which is known to mediate resistance to glucocorticoids.^10^, ^19^ It is not clear if this activation takes place in the blood or reflects the activation state of CLL cells in lymphoid organ microenvironments^20^ that are flushed into the circulation by ruxolitinib (Figure 2A).

Inclusion criteria for this trial were quite broad and included patients at almost any time in their treatment course with ibrutinib. The trial was a modification of a prior phase 1 dose‐finding study carried out in patients who exhibited an “insufficient response” to ibrutinib after at least 6 months.^4^ An inefficient response in that trial was defined essentially as any evidence of remaining adenopathy or circulating CLL cells. It was designed to identify patients with persisting leukemia cells because there is a clinical need for strategies to clear CLL cells that might ultimately cause disease progression on ibrutinib and also to shorten the time on ibrutinib for patients who might eventually enter a CR.^31^ Only modest therapeutic effects were noted in the phase 1 trial but it was felt that cytokine signaling remained a strong candidate to promote the survival of CLL cells in the presence of ibrutinib and ruxolitinib might still be exploited to sensitize them to cytotoxic drugs. DEX was chosen as the cytotoxic agent for this trial based on its historical use in CLL and preclinical studies showing CLL cells under in vitro microenvironmental conditions involving treatment with a Toll‐like receptor agonist and IL‐2 could be killed by DEX in combination with ibrutinib and ruxolitinib but not with ibrutinib alone. Based on these results, the inclusion criteria for this phase II trial were broadened to include patients earlier in their treatment with ibrutinib as it was expected that at least 3/8 such patients might exhibit CRs after as few as 8 months of exposure to ibrutinib that would be very unlikely with ibrutinib as single agent.

The negative results in the five patients treated with DEX, ruxolitinib, and ibrutinib suggest better in vitro microenvironmental models are needed to predict in vivo results.^32^ While a stronger cytotoxic agent such as venetoclax^33^ might have been more effective than DEX, the results reported here suggest addition of ruxolitinib would likely still activate CLL cells (Figure 4) and may cause resistance to BCL2 inhibition.^34^

Serum LDH was increased by ruxolitinib as a single agent in patients with high tumor burdens.^3^ Ruxolitinib‐induced changes in LDH were not appreciated in the phase I trial that involved patients with low tumor burdens.^4^ Inclusion of patients with higher tumor burdens earlier in their course of treatment with ibrutinib (Table 1) plus the presence of a control group treated only with DEX showed that ruxolitinib‐induced increases in serum LDH are not prevented completely by ibrutinib (Figure 1C). The origins of these LDH proteins are not clear. No other markers of tumor lysis were observed to suggest their release by dying leukemia cells. Perhaps, these increases reflect a higher metabolic state of CLL or other cells that have been activated by ruxolitinib. Consistent with this idea, *LDHA* transcripts tended to be upregulated in CLL cells following addition of ruxolitinib to ibrutinib (Table S1).

JAK3008 taking ibrutinib plus DEX without ruxolitinib died of invasive aspergillosis prior to cycle 5 of DEX. Increased rates of invasive fungal infections have been reported in CLL patients on ibrutinib, particularly in the first 6 months of treatment, and related to iatrogenic defects in macrophage function including decreased cytokine production.^21^ JAK3008 was on ibrutinib for 40 months (Table 1) before adding DEX. However, glucocorticoids are also associated with an increased risk of invasive fungal infections in ibrutinib‐treated patients^22^ and the significant drop in IgG levels caused by DEX (Figure 1B) may have contributed to impaired antifungal responses. Despite profound interference with cytokine signaling responses, only one other fungal infection (extrapulmonary *Blastomyces dermatitidis*) has been observed in 30 CLL patients treated locally with ruxolitinib with or without ibrutinib or DEX (Table 1).^3^, ^4^

Symptomatic CLL is accompanied by a complex aberrant cytokine network^23^ that is simplified considerably by ibrutinib.^6^ The cytokines remaining in patients on ibrutinib that are blocked by ruxolitinib to allow CLL cells to acquire a phenotype associated with cell cycle progression and NFκB activation (Figure 3) are presently unknown. Evidence for ongoing activity of type 1 and 2 interferons in CLL patients on ibrutinib has been found previously^5^ and the studies reported here suggest CLL cells experience IFN signaling while on ibrutinib in vivo that is turned off by ruxolitinib (Figure 3A). IFN signaling in the presence of ibrutinib may help to restrain CLL cells and constitute a previously unrecognized component of the therapeutic activity and toxicity profile of ibrutinib (Figure 4). Inhibition of IFN signaling may account in part for the apparent activation of CLL cells by ruxolitinib (Figure 3) and the curious platelet increases observed with ruxolitinib in the presence of ibrutinib (Figure 2A).^4^, ^24^

JAK inhibitors (JAKis) like ruxolitinib have significant therapeutic activity in cancers driven primarily by oncogenic JAK signaling such as myelofibrosis.^25^ Our results suggest JAKis should be used with caution in cancers driven by pathogenic activation of both NFκB and JAKs.^26^ By blocking inhibitory signaling through JAKs, ruxolitinib may promote NFκB signaling (Figures 3 and 4) and perhaps even tumor progression.^27^ This mechanism may explain the increased risk of aggressive B‐cell lymphomas in patients receiving single‐agent ruxolitinib for myelofibrosis.^28^ Concomitant use of NFκB signaling inhibitors with ruxolitinib may be more useful in such cancers.^29^

## CONFLICT OF INTEREST

DS reports grants from Novartis to support the submitted work and personal fees from Janssen outside the submitted work. The other authors declare no conflict of interest with respect to this work.

## ETHICAL STATEMENT

The trial was approved by the Sunnybrook Research Ethics Board and Health Canada and conducted according to the principles of the Declaration of Helsinki and the Guidelines for Good Clinical Practice. Studies involving human samples were reviewed and approved by the Sunnybrook Research Ethics Board (PIN 222‐2014).

## Supporting information

Table S1

## Data Availability

Data for this study are the property of the sponsor (DS) and the Sunnybrook Research Institute. Data and related documents to the study can be requested from the corresponding author from the date of publication. The access of data and documents will require the agreement of the sponsor. The data that supports the findings of this study are available in the supplementary material of this article.
